# Complete Chloroplast Genomes of Four Oaks from the Section *Cyclobalanopsis* Improve the Phylogenetic Analysis and Understanding of Evolutionary Processes in the Genus *Quercus*

**DOI:** 10.3390/genes15020230

**Published:** 2024-02-11

**Authors:** Ling-Ling Wang, Yu Li, Si-Si Zheng, Gregor Kozlowski, Jin Xu, Yi-Gang Song

**Affiliations:** 1 School of Ecological Technology and Engineering, Shanghai Institute of Technology, Shanghai 201418, China; llwang0615@163.com; 2 Eastern China Conservation Centre for Wild Endangered Plant Resources, Shanghai Chenshan Botanical Garden, Shanghai 201602, China; liyu980625@163.com (Y.L.); zhengsisi1228@163.com (S.-S.Z.); gregor.kozlowski@unifr.ch (G.K.); 3 Department of Biology and Botanic Garden, University of Fribourg, 1700 Fribourg, Switzerland; 4 Natural History Museum Fribourg, 1700 Fribourg, Switzerland

**Keywords:** *Quercus*, chloroplast genome, comparative genomic analysis, phylogenetic relationship, evolutionary selection pressure

## Abstract

*Quercus* is a valuable genus ecologically, economically, and culturally. They are keystone species in many ecosystems. Species delimitation and phylogenetic studies of this genus are difficult owing to frequent hybridization. With an increasing number of genetic resources, we will gain a deeper understanding of this genus. In the present study, we collected four *Quercus* section *Cyclobalanopsis* species (*Q*. *poilanei*, *Q*. *helferiana*, *Q*. *camusiae*, and *Q*. *semiserrata*) distributed in Southeast Asia and sequenced their complete genomes. Following analysis, we compared the results with those of other species in the genus *Quercus*. These four chloroplast genomes ranged from 160,784 bp (*Q*. *poilanei*) to 161,632 bp (*Q*. *camusiae*) in length, with an overall guanine and cytosine (GC) content of 36.9%. Their chloroplast genomic organization and order, as well as their GC content, were similar to those of other *Quercus* species. We identified seven regions with relatively high variability (*rps16*, *ndhk*, *accD*, *ycf1*, *psbZ—trnG-GCC*, *rbcL—accD*, and *rpl32—trnL-UAG*) which could potentially serve as plastid markers for further taxonomic and phylogenetic studies within *Quercus*. Our phylogenetic tree supported the idea that the genus *Quercus* forms two well-differentiated lineages (corresponding to the subgenera *Quercus* and *Cerris*). Of the three sections in the subgenus *Cerris*, the section *Ilex* was split into two clusters, each nested in the other two sections. Moreover, *Q*. *camusiae* and *Q*. *semiserrata* detected in this study diverged first in the section *Cyclobalanopsis* and mixed with *Q*. *engleriana* in the section *Ilex*. In particular, 11 protein coding genes (*atpF*, *ndhA*, *ndhD*, *ndhF*, *ndhK*, *petB*, *petD*, *rbcL*, *rpl22*, *ycf1*, and *ycf3*) were subjected to positive selection pressure. Overall, this study enriches the chloroplast genome resources of *Quercus*, which will facilitate further analyses of phylogenetic relationships in this ecologically important tree genus.

## 1. Introduction

Genetic resources include genes, genetic variants, and genetic complexes that control traits with actual or potential economic, environmental, scientific, or societal value [1,2]. The development of key genetic resources, especially for threatened and indicator species, and those that underpin biodiversity, is important for biological conservation [3,4]. With the advent of the genomic age, genomic resources can greatly assist cytogenetics, molecular biology, bioinformatics, evolutionary biology, and conservation biology.

Organellar genomes (mitochondrial and chloroplast DNA) are important in eukaryotes. The chloroplast is an important semiautonomous plant organelle with a complete genetic system that provides space for photosynthesis [5,6]. The availability of public chloroplast genomic resources has grown rapidly, which has helped us understand the relationships between angiosperms and all flowering plant families [7,8]. Because of the characteristics of inherited uniparentally conserved sequences, similar structures, and slower evolutionary rates, the chloroplast genome has also been shown to play an important role in taxonomy, phylogeny, phylogeography, genomics, and conservation biology [9,10,11,12].

*Quercus* (oaks) section *Cyclobalanopsis* (cycle-cup oaks) are exclusively found in East and Southeast Asia and are the dominant trees in tropical and subtropical areas with warm and humid climates [13,14]. *Cyclobalanopsis* is one of the largest sections in *Quercus,* with approximately 110 species, and has the highest proportion of threatened oaks [15]. Previous phylogenetic studies provided our understanding of evolutionary history and population divergence, and previous phylogeographic studies may provide insight into the distribution and evolution in geographic space and facilitate effective conservation and management strategies; previous conservation genetic studies focused on the genetic diversity, population structure, and endangered status of *Quercus*, providing key information into the genetic health of cycle-cup oak populations and scientific conservation plans [16,17,18,19,20,21,22,23,24,25]. While most of these studies are related to species from East Asia, the genetic resources of species from Southeast Asia are very rare. To gain a deeper understanding of the tropical cycle-cup oak species from Southeast Asia, it is necessary to exploit genetic and genomic data to explore their evolution and conservation.

In this context, we collected four cycle-cup oak species (*Q*. *poilanei*, *Q*. *helferiana*, *Q*. *camusiae*, and *Q*. *semiserrata*) that are mainly distributed in Southeast Asia. *Quercus poilanei*, *Q*. *helferiana*, and *Q*. *semiserrata* are widely distributed in Southwest China, Thailand, Laos, Vietnam, Malaysia, and Myanmar, whereas *Q*. *camusiae* is a critically endangered species distributed only in the boundary area between China and Vietnam [14]. Using next-generation sequencing data, the chloroplast (cp) genomes of four cycle-cup oak species were assembled and annotated. We investigated the typical structural characteristics, abundance of simple sequence repeats (SSRs) and large repeat sequences, and codon preferences of these four species. Combined with the cp genomes of the other 20 species in this section [25,26,27,28,29,30], we performed the following analyses: (1) comparative genomic analysis, (2) construction of the cp genomic phylogeny of section *Cyclobalanopsis*, and (3) evolutionary selection pressure analysis. In the present study, we provided cp genomic resources for these four cycle-cup oaks and resolved their structures, phylogenetic relationships, and adaptive evolution.

## 2. Materials and Methods

### 2.1. Plant Samples and DNA Extraction and Sequencing

Fresh and healthy leaf samples from the four *Quercus* section *Cyclobalanopsis* species were harvested and desiccated on silica gel (Table 1). The samples were deposited in the herbarium of the Shanghai Chenshan Botanical Garden. Total plant DNA was extracted from leaf tissues using a modified cetyl trimethyl ammonium bromide (CTAB) protocol [31]. Total genomic DNA was double-terminally sequenced using the high-throughput sequencing platform DNBSEQ. High-quality clean data were obtained by removing low-quality sequences [32].

### 2.2. Chloroplast Genome Assembly, Annotation, and Visualization

The cp genomes of the four *Quercus* section *Cyclobalanopsis* species in this study were de novo assembled using “get_organelle_from_reads.py” in GetOrganelle v1.7.6.1 software [33]. The sequences were manually checked for assembly into rings using Bandage [34]. The online annotation program GeSeq (https://chlorobox.mpimp-golm.mpg.de/geseq.html; accessed on 5 July 2023) was used to genomes annotate the .gb files for subsequent analysis [35]. Chloroplast genome maps of the four species were generated using the online program OrganellarGenomeDRAWv1.3.1 (https://chlorobox.mpimp-golm.mpg.de/OGDraw.html; accessed on 8 July 2023) [36]. The basic features of the cp genomes, including the length, guanine and cytosine (GC) content, and genes, were identified using Geneious R9.0.2 software [37].

### 2.3. Repeated Sequence Analysis

Simple sequence repeats (SSRs) were identified using the online program MIcroSAtellite (MISA, https://webblast.ipk-gatersleben.de/misa/; accessed on 15 July 2023) [38]. The repeat number thresholds from mononucleotides to hexanucleotides were set at 10, 5, 4, 3, 3, and 3. Composite microsatellites were identified by setting the minimum distance between two SSRs to be < 100 bp. The dispersed repeat sequences, including forward repeat sequences (F), reverse repeat sequences (R), complementary repeat sequences (C), and palindromic repeat sequences (P), were searched by the REPuter (https://bibiserv.cebitec.uni-bielefeld.de/reputer; accessed on 15 July 2023) [39]. The Hamming distance, maximum computed repeats, and minimal repeat size were set to 3, 50, and 30, respectively. Minisatellite repeat sequences (M) of at least 10 bp in length were identified using Tandem Repeats Finder (TRF, http://tandem.bu.edu/trf/trf.html; accessed on 15 July 2023). The alignment parameters for the matches, mismatches, and indels were set to 2, 7, and 7, respectively. The minimum alignment score and maximum period size were set to 80 and 500, respectively [40,41].

### 2.4. Codon Usage Bias Analysis

The coding sequences (CDS) were extracted using Geneious R9.0.2 software and screened on the condition that ATG was the starting codon and the sequence length was ≥ 300 bp. We also calculated the codon usage bias parameters, including codon base content, effective number of codons (ENC), and relative synonymous codon usage (RSCU), using CodonW1.4.2, with default parameters. The RSCU analysis was performed using R and the ENC-plot, PR2-bias-plot, and neutrality-plot analyses were performed using Origin2021 [42,43].

### 2.5. Comparative Genome Analyses of Chloroplast Genomes

The Mauve plugin in Geneious R9.0.2 software with default parameters was used to determine whether structural changes existed in the cp genomes of the 20 *Quercus* section *Cyclobalanopsis* species. IRscope was used to map the genetic structure of the boundary regions between inverted repeat (IR) and single copy (SC) regions [44]. Using the cp genome of *Q*. *acuta* as the reference sequence, alignments of 20 *Quercus* section *Cyclobalanopsis* species were visualized using the cp comparative genomics tool mVISTA (http://genome.lbl.gov/vista/mvista/submit.shtml; accessed on 25 July 2023) [45]. Complete cp genomes from 20 *Quercus* section *Cyclobalanopsis* species were aligned using the multiple sequence alignment program MAFFT v7.487 [46]. Sliding window analysis was performed using DnaSP v6.12.03 software [47], with a step size of 200 bp and window length of 800 bp, to calculate nucleotide diversity (Pi values) and detect highly variant hotspots in the cp genomes [48].

### 2.6. Phylogenetic Analysis

To establish phylogenetic relationships, a phylogenetic tree of *Quercus* was constructed using maximum likelihood (ML) method based on 33 complete cp genomes [49]. *Fagus engleriana* and *Juglans mandshurica* were used as outgroup species. MAFFT v7.487 was used to align the complete cp genomes of 33 species [46]. Next, the phylogenetic tree was reconstructed using IQ-tree v2.1.3 [50]. The ML tree adopted TVM + F + R2 as the best nucleotide replacement model with 1000 bootstrap replicates [51]. Finally, the constructed phylogenetic tree was further edited and visualized using FigTree v.1.4.4 (http://tree.bio.ed.ac.uk/software/figtree/; accessed on 5 August 2023).

### 2.7. Evolutionary Selection Pressure Analysis

To identify the evolutionary selection pressure in the cp genomes of the *Quercus* section *Cyclobalanopsis* [52], non-synonymous (Ka) and synonymous (Ks) ratios (Ka/Ks) were calculated using the Codeml program in the PAML v4.9j software package [53]. The Codeml program requires four files to complete the run: the program file, configuration file, and alignment sequence files and phylogenetic tree files. The four types of files were placed in the same directory and the selection pressure of the 79 common protein coding genes (PCGs) was identified using the site model. Six models (seqtype = 1, model = 0, and NSsites = 0, 1, 2, 3, 7, and 8) were used to detect the potential sites of positive selection. The likelihood ratio test (LRT) was performed after pairwise comparisons of three pairs of models: M0 (single-ratio) vs. M3 (discrete), M1 (near-neutral) vs. M2 (positive selection), and M7 (β) vs. M8 (β and ω) [54]. Genes with *p*-values < 0.05 were selected as positive selection genes [55]. Finally, the posterior probability of sites was calculated based on Bayes empirical Bayes (BEB) to assess the significance of positively selected sites (*p* > 95%) [53].

## 3. Results

### 3.1. Chloroplast Genome Structures and Features of the Four Quercus Section Cyclobalanopsis Species

**Figure 1:**
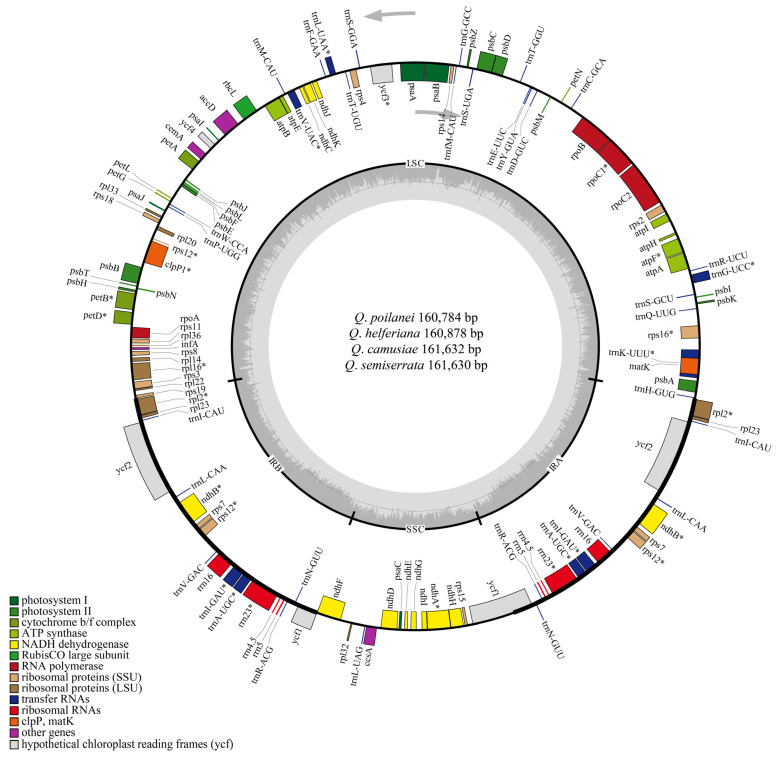
The chloroplast genome map of four *Quercus* section *Cyclobalanopsis* species. The outermost circle is the genes annotated in the chloroplast genome. Genes outside the circle are transcribed in the counterclockwise direction, whereas those inside the circle are transcribed in the clockwise direction. Different colored genes refer to different functions. The length and boundary of the LSC, SSC, and two IRs are indicated in the inner circle. The dark gray area indicates GC content while the lighter gray corresponds to the AT content of the genome. The gray arrows represent that sequences are selected in a forward direction. “*” represents that gene has intron.

The length of the four assembled cp genomes ranged from 160,784 bp in *Q*. *poilanei* to 161,632 bp in *Q*. *camusiae*. All four species exhibited a typical circular tetrad structure, including two single copy regions (large single copy (LSC) and small single copy (SSC)) and two inverted repeat regions (IRs) with similar lengths in the same regions (Figure 1 and Table 2). The total GC content was 36.9% of four *Quercus* section *Cyclobalanopsis* species. In addition, the GC content differed slightly among the different regions of these four species, and the GC content in the IR region was significantly higher than that in the LSC and SSC regions (Table 2).

All four cp genomes encode 131 genes, including 86 PCGs, 37 transfer RNA genes (tRNAs), and 8 ribosomal RNA genes (rRNAs) (Table 2). The names, numbers, and orders of the genes annotated in the cp genomes were consistent among the four species. We found that 83 genes were located in the LSC region (including 61 PCGs and 22 tRNAs) and 12 genes were located in the SSC region (including 11 PCGs and 1 tRNA). The two IR regions contained 18 duplicate genes, including 7 PCGs (*rps12*, *rps7*, *rpl2*, *rpl23*, *ndhB*, *ycf1*, and *ycf2*), 7 tRNAs (*trnA-UGC*, *trnI-GAU*, *trnL-CAA*, *trnI-CAU*, *trnN-GUU*, *trnV-GAC*, and *trnR-ACG*), and 4 rRNAs genes (*rrn4.5S*, *rrn5S*, *rrn16S,* and *rrn23S*) (Table 3). Except for *ycf1* and *rps12*, all other genes were located in a single region, while *ycf1* genes spanned the IRs and SSC regions, and *rps12* spanned the IRa and LSC regions (Figure 1).

### 3.2. Repeated Sequences Analysis of Four Quercus Section Cyclobalanopsis Species

**Figure 2:**
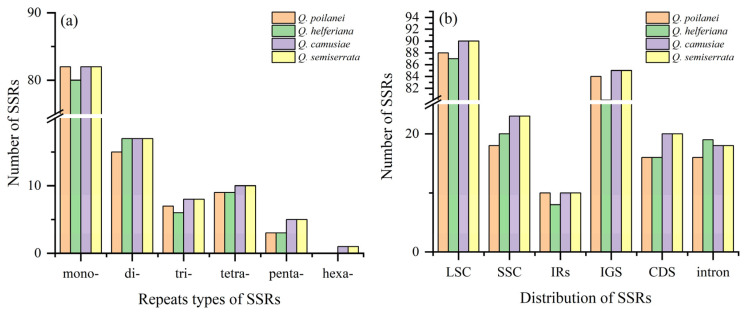
The number and distribution of SSRs of four *Quercus* section *Cyclobalanopsis* chloroplast genomes: (**a**) The number of SSRs with six repeat types (mononucleotides: mono-, dinucleotides: di-, trinucleotides: tri-, tetranucleotides: tetra-, pentanucleotides: penta-, and hexanucleotides: hexa-) and (**b**) The number of SSRs in different regions of chloroplast genomes.

The total number of SSRs identified in the cp genomes of the four *Quercus* section *Cyclobalanopsis* species was 477, ranging from 115 in *Q*. *helferiana* to 123 in *Q*. *semiserrata*. The number of the same type of SSR showed only slight variation among the four species (80–82 mononucleotides, 15–17 dinucleotides, 6–8 trinucleotides, 9–10 tetranucleotides, 3–5 pentanucleotides, and 0–1 hexanucleotides) (Figure 2a and Table S1). The main types of SSRs were mononucleotides and dinucleotides, which account for 80% of the total. The mononucleotides type was the largest, especially the A/T base type, which was far higher than that of the other types (Table S1). Additionally, the distribution of SSRs in the LSC region (74.4%) was higher than that in the IR (8%) and SSC regions (17.6%). The distribution of SSRs in intergenic spacer (IGS) regions (70%) was also higher than that in the CDS (15.1%) and intron regions (14.9%) (Figure 2b and Table 4).

**Figure 3:**
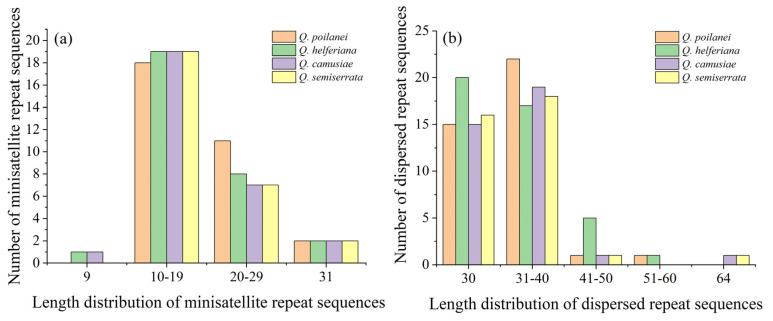
The length distribution of dispersed repeat sequences: minisatellite repeat sequences (**a**) and minisatellite repeat sequences (**b**) of four *Quercus* section *Cyclobalanopsis* species.

In total, 154 dispersed repeat sequences (D) were identified among the four cp genomes, ranging from 36 in *Q*. *semiserrata* to 43 in *Q*. *helferiana*. Meanwhile, 14–18 were forward repeat (F), 2 or 3 were reverse repeat (R), and 19–23 were palindromic repeat (P) sequences. Only one complementary repeat sequence (C) was identified in *Q*. *poilanei*. The lengths of the dispersed repeat sequences ranged from 30 to 64 bp and were concentrated between 30 and 40 bp (Figure 3a and Table 5). Finally, 117 minisatellite repeat sequences (M) were identified in the four chloroplast genomes, ranging from 28 in *Q*. *semiserrata* and *Q*. *camusiae* to 31 in *Q*. *poilanei*. The copy number of the minisatellite repeat sequences was mainly between 2 and 4, and the length distribution was concentrated between 10 and 19 bp in the four *Quercus* section *Cyclobalanopsis* species (Figure 3b and Table 5).

### 3.3. Codon Usage Bias Analysis of Four Quercus Section Cyclobalanopsis Species

**Figure 4:**
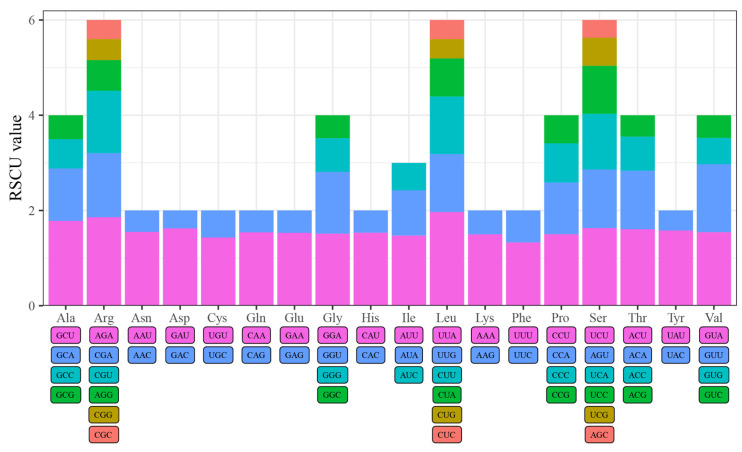
The RSCU analysis of chloroplast genomes of four *Quercus* section *Cyclobalanopsis* species.

Codon usage bias analysis was performed on 50 CDS selected from these four species. We found that the GC content at the first codon site was the highest, while that at both the second and third sites was less than 50%. Moreover, there was a decreasing trend in GC1 > GC2 > GC3, further indicating that the chloroplast genomes were rich in A/T (Table S3). All amino acids are encoded by two to six codons, except for methionine (Met), which is encoded by the ATG codon, and tryptophan (Trp), which is encoded by the TGG codon. Among the 59 synonymous codons with relative synonymous codon usage (RSCU) values, 30 high-frequency codons with an RSCU > 1 ended in A/U, whereas the remaining 29 were low-frequency codons with an RSCU < 1 (Figure 4 and Table S3). The codon with the largest RSCU value was UUA, which encodes leucine (Leu), followed by AGA, which encodes arginine (Arg) (Figure 4).

**Figure 5:**
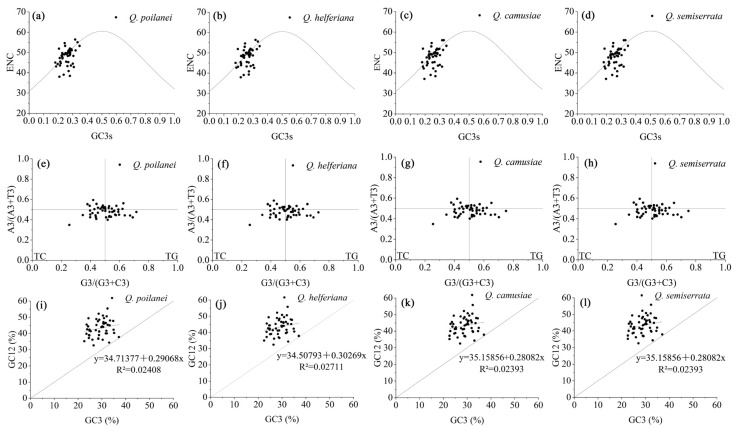
ENC-plot analysis (**a**–**d**), PR2-bias-plot analysis (**e**–**h**), and neutrality-plot analysis (**i**–**l**) of chloroplast genomes of four *Quercus* section *Cyclobalanopsis* species.

In the three analyses of the factors affecting codon preference, we found that codon preference in chloroplast genomes was the result of base mutations, natural selection, and other factors (Figure 5). In the ENC-plot analysis, most genes were distributed along or near the standard curve, indicating that codon preference was mainly affected by base mutations. However, a few genes deviated and fell far below the standard curve, indicating that the codon preference was influenced by natural selection (Figure 5a–d). In the PR2-bias-plot analysis, the four bases at the third codon site were unevenly distributed within the four areas divided by the vertical lines from the central point. The third site of the codon preferred to use base T over base A, while the numbers of bases G and C were similar at these sites. The analysis showed that codon preference in chloroplast genomes was formed by multiple factors, including base mutations and natural selection (Figure 5e–h). In the neutrality-plot analysis, GC12 and GC3 values were positively correlated with non-significance, suggesting that codon preference in the chloroplast genomes was more affected by natural selection than by base mutations (Figure 5i–l).

### 3.4. Comparative Genome of Chloroplast Genomes of Quercus Section Cyclobalanopsis

In this study, we used the Mauve plugin in Geneious R9.0.2 software to determine the differences between the chloroplast genomes of 20 *Quercus* section *Cyclobalanopsis* species. Multiple alignment analysis showed that the genome structure and gene arrangement were consistent and that there were no gene rearrangements or inversions with a good collinearity relationship (Figure S1). Therefore, the Mauve alignment further illustrated the high conservation of the 20 chloroplast genomes of *Quercus*.

**Figure 6:**
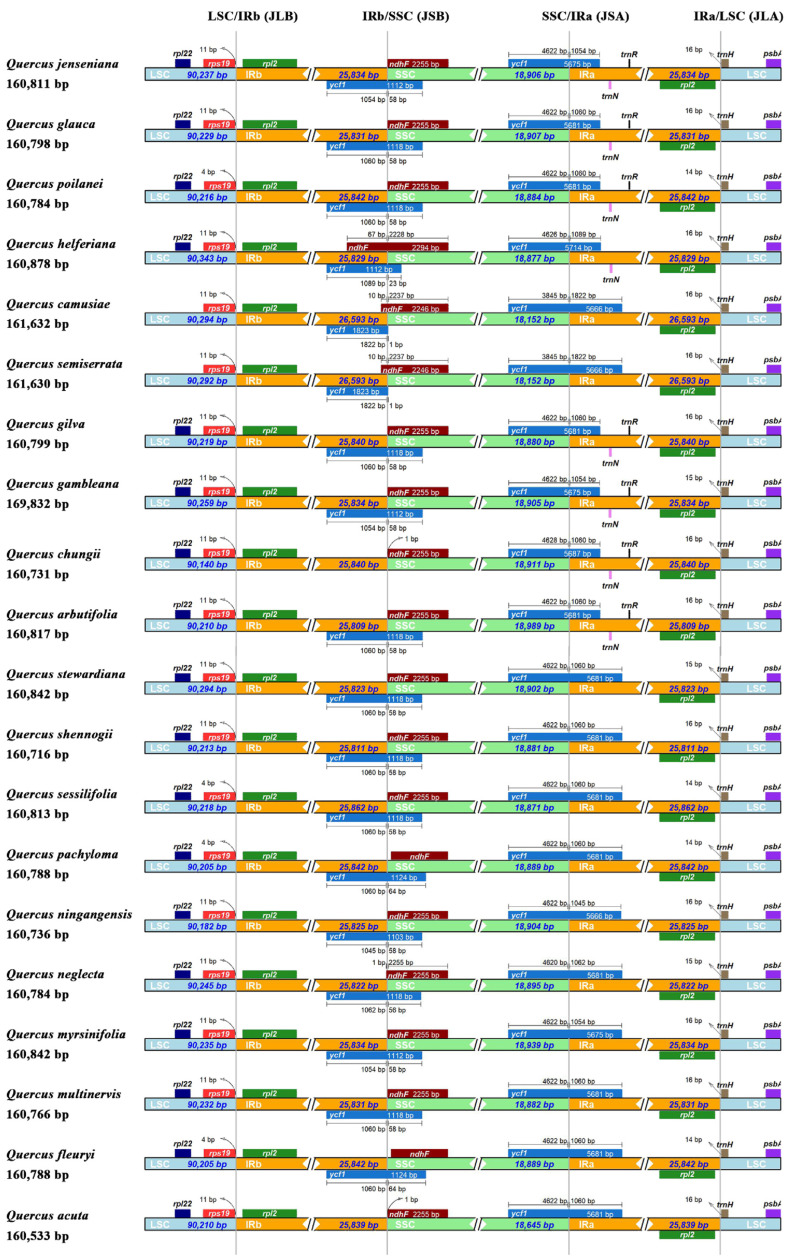
Comparison of the junction regions (JLA, JLB, JSB, and JSA) among 20 chloroplast genomes of *Quercus* section *Cyclobalanopsis* species. Genes are denoted by colored boxes. The numbers above the gene boxes indicates the distance between the end of the gene and the border sites.

The results of the contraction and expansion of the IR regions indicated that although the genome structure and size were highly conserved in the 20 chloroplast genomes, the boundary regions between the IR and LSC/SSC regions still varied slightly. The junction region of the LSC and IRb (JLB) lies in the IGS between *rps19* and *rpl2*. The *rps19* gene of most *Quercus* section *Cyclobalanopsis* species had an 11 bp shift at the JLB boundary, but *Q*. *poilanei*, *Q*. *sessilifolia*, and *Q*. *pachyloma* expanded to only a 4 bp shift. The *ndhF* gene of most *Quercus* section *Cyclobalanopsis* species was located in the SSC region, whereas different levels contracted to the IRb region in *Q*. *helferiana*, *Q*. *camusiae*, *Q*. *semiserrata*, and *Q*. *neglecta*. Specifically, the two junction regions between IRa/IRb and SSC (JSA and JSB) were located in two *ycf1* genes. The *ycf1* gene located in JSA varied between 1045 and 1089 bp in the IRa region and between 3845 and 4628 bp in the SSC region. However, the *ycf1* gene located in JSB varied between 1045 and 1822 bp in the IRb region and only from one to 64 bp in the SSC region (Figure 6).

We used mVISTA to perform sequence variability analysis using *Q*. *acuta* as the reference genome. The results showed a high sequence similarity, where the non-coding and SC regions exhibited higher levels of differentiation than the coding and IR regions among the 20 chloroplast genomes of cycle-cup oaks. Overall, the *ycf1* gene was particularly different among the 20 chloroplast genomes, and the sequence similarity of *ycf1* gene was < 50% in the three species of *Q*. *fleuryi*, *Q*. *glauca*, and *Q*. *pachyloma*. Moreover, the exon regions of two PCGs (*ndhF* and *ycf1*) and the conserved non-coding regions of three IGS (*petN—psbM*, *psbZ—trnG-UCC*, and *rpl32—trnL-UAG*) showed high variability (Figure S2).

**Figure 7:**
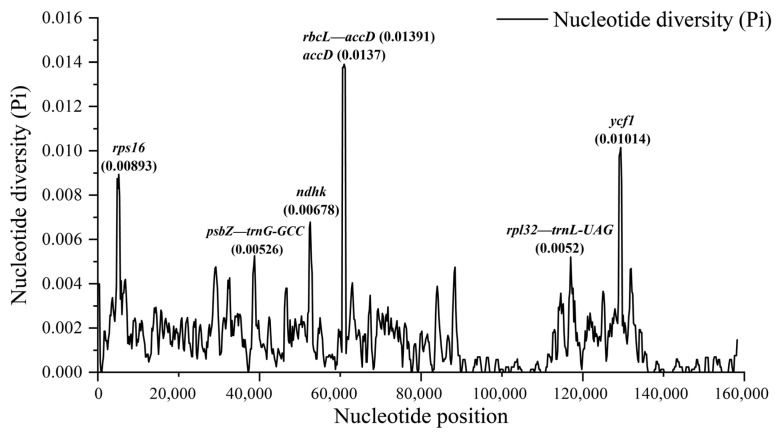
Sliding window analysis of the 20 chloroplast genomes of *Quercus* section *Cyclobalanopsis*. The X-axis represents nucleotide positions of the middle point of the window and the Y-axis represents the value of nucleotide diversity (Pi) per window.

Sliding window analysis was performed using the DnaSP software to calculate nucleotide diversity values (Pi) among all chloroplast genomes. The results indicated that the Pi value in the chloroplast genomes of *Quercus* section *Cyclobalanopsis* ranged from 0 to 0.01391, with an average of 0.00149. We found seven highly divergent regions (Pi > 0.005), four of which were located in the PCGs (*rps16*, *ndhk*, *accD*, and *ycf1*) and three in the IGS (*psbZ—trnG-GCC*, *rbcL—accD*, and *rpl32—trnL-UAG*) (Figure 7). These results could potentially provide plastid markers for further taxonomic and phylogenetic studies of *Quercus*.

### 3.5. Phylogenetic Relationships

**Figure 8:**
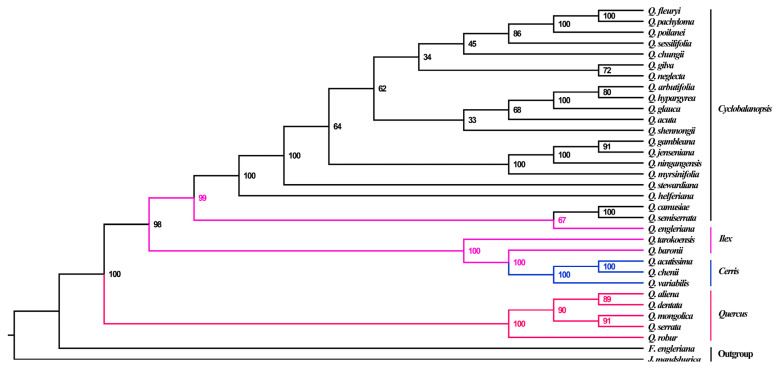
The phylogenetic tree among 33 of the chloroplast complete genomes based on the ML method. Values beside the branch represented bootstrap support (BS). Abbreviations: *Quercus* (*Q*.), *Fagus* (*F*.), and *Juglans* (*J*.).

With respect to the ML approach, phylogenetic relationships were reconstructed based on the whole chloroplast genomes of the four species sequenced in this study and closely related species in the *Quercus* genus. The whole chloroplast genomes of the 31 *Quercus* species from four sections and two outgroups (*F. engleriana* and *J. mandshurica*) were aligned. The results indicated that 31 species of *Quercus* were clearly differentiated into two clades with high bootstrap support values (Figure 8). *Quercus* belonging to the subgenus *Quercus* formed one clade, whereas the other three sections belonging to the subgenus *Cerris* formed another clade. Of the three sections in the subgenus *Cerris*, the section *Ilex* split into two clusters, each nested with the other two sections. *Quercus camusiae* and *Q*. *semiserrata* detected in this study diverged first in the section *Cyclobalanopsis* and mixed with *Q*. *engleriana* from the section *Ilex*. Followed this cluster, *Q. helferiana* was differentiated alone. The section *Cyclobalanopsis* was divided into two major evolutionary clusters, in which *Q*. *poilanei* was also located (Figure 8).

### 3.6. Selection Pressure Analysis

In the present study, a site model of the PAML program was used to detect the selection pressure of common PCGs in the chloroplast genomes of 20 *Quercus* section *Cyclobalanopsis* species. A total of 28 and 33 genes with positive selection sites were identified in M2 and M8, respectively. Based on pairwise comparisons of M0 vs. M3, M1 vs. M2, and M7 vs. M8, 33 PCGs with positive selection sites were subjected to the likelihood ratio test (LRT). Genes with a significance of *p* < 0.05 were selected as positive selection sites. The results showed that a total of 11 PCGs underwent positive selection (*atpF*, *ndhA*, *ndhD*, *ndhF*, *ndhK*, *petB*, *petD*, *rbcL*, *rpl22*, *ycf1*, and *ycf3*). Based on the Bayesian empirical Bayes algorithm (BEB) analyses in model M8, 103 sites showed positive selection among the 11 PCGs, 24 of which showed significant positive selection (Table 6 and Table S4).

## 4. Discussion

### 4.1. Architecture of Chloroplast Genomes in Quercus Section Cyclobalanopsis

In this study, we successfully assembled the chloroplast genomes of four *Quercus* section *Cyclobalanopsis* species. The size of the four chloroplast genomes (~160 kb) corresponded to that of photosynthetic land plants, which vary in size from 120 to 170 kb [56]. Similar to the chloroplast genome structure of other *Quercus* species, we found that the chloroplast genomes of *Quercus* section *Cyclobalanopsis* are highly conserved with a typical circular tetrad structure [25,27,30,57]. The overall GC content was not distinct among the four species, but the IR regions had a significantly higher GC content than the SC regions owing the presence of unique rRNA genes [30,58]. Genome annotation revealed that the number, order, and function of genes were also highly conserved in *Quercus* section *Cyclobalanopsis*.

Nonetheless, the IR regions are important for stabilizing the chloroplast structure. The expansion and contraction of IRs regions are the main factors influencing the length of chloroplast genomes in different species [59]; therefore, they are of great significance for evolutionary research [60]. Differences in the four boundary regions among species frequently lead to further changes in chloroplast genome size [61]. In the present study, the distribution of the boundary genes in the four regions was conserved, except for a slight difference in *ndhF* in JSB. Most of the compared species of *Quercus* section *Cyclobalanopsis* found no significant expansion or contraction in the IR regions, as the same conditions with other *Quercus* species [25,27,62].

Repeat sequences are widespread in plant genomes and play important roles in the heredity, variation, and evolution of genomes [63,64,65]. We identified simple sequence repeat (SSRs), dispersed repeat sequences (D), and minisatellite repeat sequences (M) in the chloroplast genomes of four *Quercus* species. The results showed that the detected repeats were essentially composed of A and T bases with a strong A/T preference, which is consistent with previous findings [26,29,66]. Moreover, most of the repeat sequences were located in the LSC and IGS regions, which is consistent with the findings of previous studies [25,27,29]. As effective molecular markers, SSRs have been extensively studied in discrimination, breeding, conservation, and phylogenetic studies at both the species and population level [67,68,69].

Codon usage bias is an important evolutionary feature that is prevalent in biological taxa and subject to natural selection, base mutations, and other factors [70,71]. The GC content at the first, second, and third codon sites in the chloroplast genomes showed a decreasing trend of GC1 > GC2 > GC3. The GC content is the main factor responsible for codon usage bias and may play an important role in the evolution of genome structure [72]. The chloroplast genomes of the four *Quercus* section *Cyclobalanopsis* species had a relatively weak codon preference. A total of 30 of the 59 synonymous codons had RSCU values > 1 and ended with A/U. From the RSCU value and GC content, the third codon site was biased towards A/U, which is common in angiosperms [6,73].

The chloroplast genomes of 20 species in *Quercus* section *Cyclobalanopsis* were subjected to comparative genomic analyses to study the differences between them. The results showed differences in variation between the regions of the chloroplast genomes. The variation in the SC regions was higher than that in the IR regions, whereas that in the IGS regions was higher than that in the coding regions. In addition, the regions of high variability detected in this study can be used for DNA barcoding and species identification and classification [74,75].

### 4.2. Phylogeny and Evolution of the Quercus Chloroplast Genome

As a species-rich, widely distributed, and long-lived genus, *Quercus* is a hotspot plant for phylogenetic research [76,77,78,79,80,81]. Due to complex evolutionary issues such as convergent evolution, extensive introgressive hybridization, and incomplete lineage classification, the phylogenetic/phylogenomic studies of *Quercus* have received significant attention from botanists [82,83,84]. Therefore, we performed a phylogenetic analysis of *Quercus* species using four new complete chloroplast genomes from cycle-cup oaks.

Based on restriction site-associated DNA sequencing of nuclear DNA, *Quercus* subgenus *Cerris* is divided into three recognized sections: *Cyclobalanopsis*, *Cerris*, and *Ilex* [85,86]. The chloroplast phylogenomics in previous studies supported the nesting of the *Cerris* and *Cyclobalanopsis* sections in section *Ilex* [24,29]. Notably, *Quercus* section *Ilex* was paraphyletic, and the section *Cerris* nested into the first branch of Section *Ilex*. Except for *Q. poilanei*, the other three species in this study were located at the base in section *Cyclobalanopsis*. Incomplete lineage classification or introgression between the ancestral lineages in these three sections plays an important role in shaping the current relationships. In addition, oaks are actually considered typical hybrid species [85]. Overall, this study greatly enriches the chloroplast genome resources of *Quercus*, which provides convenience for further analysis of phylogenetic and internal genetic relationships.

At the chloroplast genome level, we found that 11 PCGs had undergone positive selection in the *Quercus* section *Cyclobalanopsis*. Among these, the *ycf1* gene was found to have the most sites under positive selection; however, the possible evolutionary significance of this result remains to be elucidated owing to the uncertainty of the function of this gene. The *atpF* gene encodes a subunit of H+-ATP synthase, which is required for electron transport and photophosphorylation during photosynthesis [87]. The adaptive evolution of *atpF* may affect the chloroplast energy metabolism [88]. Positive selection was detected in four *ndh* genes (*ndhA*, *ndhD*, *ndhF*, and *ndhK*) whose adaptive evolution may influence energy conversion and resistance to photooxidative stress [89,90]. Notably, the *ndh* genes were lost or pseudogenized in many gymnosperms [91]. The *rbcL* gene plays an important role in photosynthesis and is subject to positive selection in many higher plants [92]. Furthermore, *petD* and *petB* also underwent positive selection; however, more evidence is needed to confirm their evolutionary significance. Some researchers have found that *petD* gene mutation can reduce the photosynthetic rate of chlamydomonas [93]. Our identification of positively selected genes in this analysis could lead to a better understanding of the evolution of *Quercus* species.

## Figures and Tables

**Table 1:** Basic information about four *Quercus* section *Cyclobalanopsis* species in this study.

Species	Voucher No.	GenBank Accession No.	Latitude (N)	Longitude (E)	Place of Collection
*Q. poilanei*	DM15650	OR835153	23.416667	108.36667	Daming Mountain, China
*Q. helferiana*	DM19757	OR835154	18.495611	99.302050	Kun Tan National Park, Thailand
*Q. camusiae*	DM19880	OR966887	18.539589	98.534078	Mae Klang Luang Trail, Thailand
*Q. semiserrata*	DM19890	OR966888	18.541483	98.543278	Mae Klang Luang Trail, Thailand

**Table 2:** Complete chloroplast genome structures and features of four *Quercus* section *Cyclobalanopsis* species. Abbreviations: LSC (Large Single Copy), SSC (Small Single Copy), IR (Inverted Repeat), GC (guanine and cytosine), PCGs (Protein coding genes), tRNA (Transfer RNA gene), and rRNA (Ribosomal RNA gene).

Species	*Q. poilanei*	*Q. helferiana*	*Q. camusiae*	*Q. semiserrata*
Genome size (bp)	160,784	160,878	161,632	161,630
Length of LSC (bp)	90,216	90,343	90,294	90,292
Length of IRs (a/b) (bp)	25,842	25,829	26,593	26,593
Length of SSC (bp)	18,884	18,877	18,152	18,152
Total GC content (%)	36.9	36.9	36.9	36.9
GC content of LSC (%)	34.74	34.74	34.75	34.75
GC content of IRs (%)	42.77	42.70	42.35	42.35
GC content of SSC (%)	31.11	31.11	31.22	31.22
Number of genes	131	131	131	131
Number of PCGs	86	86	86	86
Number of tRNAs	37	37	37	37
Number of rRNAs	8	8	8	8

**Table 3:** Gene classification of the chloroplast genomes of four *Quercus* section *Cyclobalanopsis* species. Genes marked with the * or ** sign are the genes with single or double introns, respectively. The duplicated genes located in IR regions are marked as (×2).

Category	Gene Group	Gene Name
Photosynthesis	Photosystem I	*psaA*, *psaB*, *psaC*, *psaI*, *psaJ*
	Photosystem II	*psbA*, *psbB*, *psbC*, *psbD*, *psbE*, *psbF*, *psbH*, *psbI*, *psbJ*, *psbK*, *psbL*, *psbM*, *psbN*, *psbT*, *psbZ*
	NADH dehydrogenase	ndhA*, ndhB*(×2), *ndhC*, *ndhD*, *ndhE*, *ndhF*, *ndhG*, *ndhH*, *ndhI*, *ndhJ*, *ndhK*
	Cytochrome b/f complex	*petA*, petB*, petD*, *petG*, *petL*, *petN*
	ATP synthase	*atpA*, *atpB*, *atpE*, atpF*, *atpH*, *atpI*
	Rubisco of Large subunit	*rbcL*
Transcription and translation	Translation initiation factor	*infA*
	Ribosomal Proteins (LSU)	*rpl14*, rpl16*, rpl2*(×2), *rpl20*, *rpl22*, *rpl23*(×2), *rpl32*, *rpl33*, *rpl36*
	Ribosomal Proteins (SSU)	*rps11*, rps12**(×2), *rps14*, *rps15*, rps16*, *rps18*, *rps19*, *rps2*, *rps3*, *rps4*, *rps7*(×2), *rps8*
	RNA polymerase	*rpoA*, *rpoB*, rpoC1*, *rpoC2*
	Ribosomal RNAs	*rrn16*(×2), *rrn23*(×2), *rrn4.5*(×2), *rrn5*(×2)
	Transfer RNAs	trnA-UGC*(×2), *trnC-GCA*, *trnD-GUC*, *trnE-UUC*, *trnF-GAA*, *trnG-GCC*, trnG-UCC*, *trnH-GUG*, *trnI-CAU*(×2), trnI-GAU*(×2), trnK-UUU*, *trnL-CAA*(×2), trnL-UAA*, *trnL-UAG*, *trnM-CAU*, *trnN-GUU*(×2), *trnP-UGG*, *trnQ-UUG*, *trnR-ACG*(×2), *trnR-UCU*, *trnS-GCU*, *trnS-GGA*, *trnS-UGA*, *trnT-GGU*, *trnT-UGU*, *trnV-GAC*(×2), trnV-UAC*, *trnW-CCA*, *trnY-GUA*, *trnfM-CAU*
Biosynthesis	Maturase	*matK*
	ATP-dependendent Protease	clpP1**
	Acetyl-CoA carboxylase	*accD*
	Envelope membrane protein	*cemA*
	C-type cytochrome synthesis gene	*ccsA*
Unknown	Conserved hypothetical chloroplast ORF	*ycf1*(×2), *ycf2*(×2), *ycf3* **, *ycf4*

**Table 4:** Distribution and number (proportion) of simple sequence repeats (SSRs) of four *Quercus* section *Cyclobalanopsis* species. Abbreviations: IGS (intergenic spacer) and CDS (coding sequence).

Species	No. (Proportion) of SSRs	Distribution of SSRs					
		LSC	SSC	IRs	IGS	CDS	Intron
*Q. poilanei*	116 (24.32%)	88	18	10	84	16	16
*Q. helferiana*	115 (24.10%)	87	20	8	80	16	19
*Q. camusiae*	123 (25.79%)	90	23	10	85	20	18
*Q. semiserrata*	123 (25.79%)	90	23	10	85	20	18
Total	477 (100%)	355 (74.4%)	84 (17.6%)	38 (8%)	334 (70%)	72 (15.1%)	71 (14.9%)

**Table 5:** The number and length distribution of minisatellite repeat sequences and dispersed repeat sequences of four *Quercus* section *Cyclobalanopsis* species. Abbreviations: M (minisatellite repeat sequence), D (dispersed repeat sequence), F (forward repeat sequence), R (reverse repeat sequence), C (complementary repeat sequence), and P (palindromic repeat sequence).

Species	No. of Repeat Sequences					Length Distribution of M				Length Distribution of D				
	M	F	R	P	C	9	10–19	20–29	31	30	31–40	41–50	51–60	64
*Q. poilanei*	31	14	3	21	1	0	18	11	2	15	22	1	1	0
*Q. helferiana*	30	18	2	23	0	1	19	8	2	20	17	5	1	0
*Q. camusiae*	28	15	2	19	0	1	19	7	2	15	19	1	0	1
*Q. semiserrata*	28	15	2	19	0	0	19	7	2	16	18	1	0	1
Total	117	62	9	82	1	2	75	33	8	66	76	8	2	2

**Table 6:** Likelihood ratio test (LRT) and positive selection sites under different site models of PCGs of four *Quercus* section *Cyclobalanopsis* (Taking *atpF* for example). “**” represents a value of positively selected sites greater than 99%.

Gene	*atpF*		
Model Comparison	M0 vs. M3	M1 vs. M2	M7 vs. M8
df	4	2	2
ΔlnL	36.484478	21.965641	22.022258
2ΔlnL	72.968956	43.931282	44.044516
LRT (*p*-value)	5.35604 × 10^−15^	2.88698 × 10^−10^	2.72807 × 10^−10^
Positively selected sites	/	17A (0.621), 49S (0.996 **), 50D (0.993 **), 52N (0.994 **), 54R (1.000 **), 104N (0.545)	17A (0.674), 49S (0.998 **), 50D (0.997 **), 52N (0.998 **), 54R (1.000 **), 104N (0.598)

## Data Availability

The data that support the finding of this study are openly available in the GenBank of NCBI at https://www.ncbi.nlm.nih.gov (accessed on 15 July 2023), reference number (OR835153).

## Supplementary Materials

The following supporting information can be downloaded at: https://www.mdpi.com/article/10.3390/genes15020230/s1. Figure S1: Mauve alignment of 20 chloroplast genomes of *Quercus* section *Cyclobalanopsis*. The box structure below the genome represents the corresponding gene annotation information: the white rectangle represents PCGs, the red rectangle represents rRNAs, and the green rectangle represents tRNAs. The introns are connected by line segments; Figure S2: Sequence alignment of the chloroplast genomes of four *Quercus* section *Cyclobalanopsis* species. The *Q*. *acuta* was used as reference. The gray arrow above the map shows the location of the reference sequence gene, and the direction of the arrow is the forward or reverse direction of the gene. The position of the genome is shown on the horizontal axis at the bottom of each block. The alignment similarity percentages are shown on the right side of the map (vertical axis). Genome regions are color coded as exon, UTR, mRNA, and conserved non-coding sequences (CNS); Table S1: Simple sequence repeats (SSRs) number in the chloroplast genomes of four *Quercus* section *Cyclobalanopsis* species. Abbreviations: LSC (Large Single Copy), SSC (Small Single Copy), IRs (Inverted Repeats), IGS (Intergenic Spacer), and GR (Gene Region); Table S2a: Codon parameter characterization of chloroplast genome of *Q*. *poilanei*. Abbreviations: ENC (Effective Number of Codon); Table S2b: Codon parameter characterization of chloroplast genome of *Q*. *helferiana*. Abbreviations: ENC (Effective Number of Codon); Table S2c: Codon parameter characterization of chloroplast genome of *Q. camusiae* and *Q*. *semiserrata*. Abbreviations: ENC (Effective Number of Codon); Table S3: The relative synonymous codon usage in four chloroplast genomes of *Quercus* section *Cyclobalanopsis*; Table S4: Likelihood ratio test (LRT) and positive selection sites under different site models of PCGs of four *Quercus* section *Cyclobalanopsis*.
