# Supplementary material for: Complete Chloroplast Genomes of Four Oaks from the Section Cyclobalanopsis Improve the Phylogenetic Analysis and Understanding of Evolutionary Processes in the Genus Quercus
Source: Genes (Basel). 2024 Feb 11;15(2):230. doi: 10.3390/genes15020230 (PMC10888318; doi:10.3390/genes15020230)
Supplement: Supplementary file 1 [file genes-15-00230-s001.zip › genes-2835175-supplementary.pdf]

Complete Chloroplast Genomes of Four Oaks from the Section *Cyclobalanopsis* Improve the Phylogenetic Analysis and Understanding of Evolutionary Processes in the Genus *Quercus*

Ling-Ling Wang [1,2,†], Yu Li [2,†], Si-Si Zheng [2], Gregor Kozlowski [2,3,4], Jin Xu [1,*] and Yi-Gang Song [2,*]

[1] School of Ecological Technology and Engineering, Shanghai Institute of Technology, Shanghai 201418, China; llwang0615@163.com
[2] Eastern China Conservation Centre for Wild Endangered Plant Resources, Shanghai Chenshan Botanical Garden, Shanghai 201602, China; liyu980625@163.com (Y.L.); zhengsisi1228@163.com (S.-S.Z.); gregor.kozlowski@unifr.ch (G.K.)
[3] Department of Biology and Botanic Garden, University of Fribourg, 1700 Fribourg, Switzerland
[4] Natural History Museum Fribourg, 1700 Fribourg, Switzerland
* Correspondence: xujin@sit.edu.cn (J.X.); ygsong@cemps.ac.cn (Y.-G.S.)
† These authors contributed equally to this work.

Supplementary

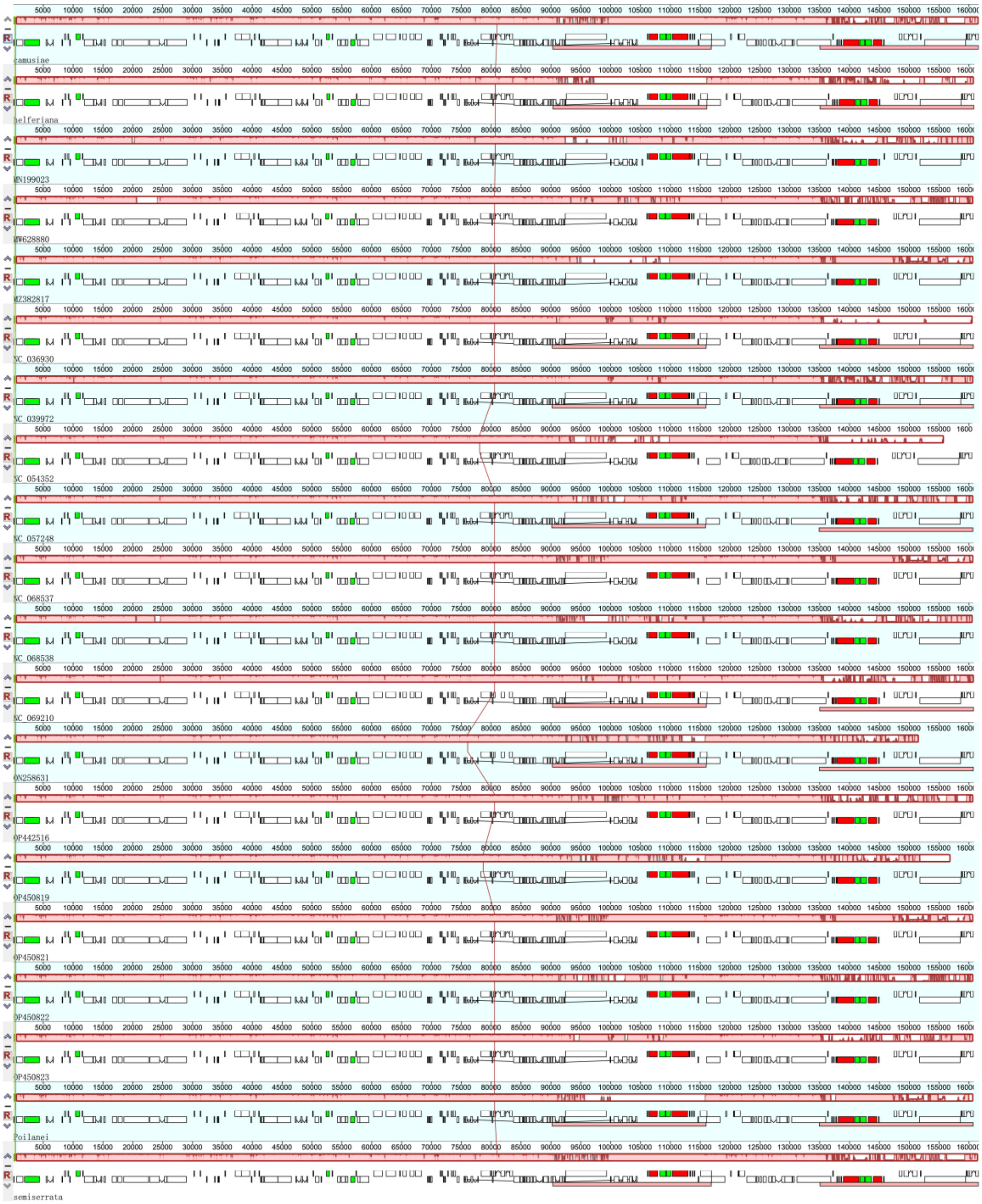

Figure S1. Mauve alignment of 20 chloroplast genomes of *Quercus* section *Cyclobalanopsis*. The box structure below the genome represents the corresponding gene annotation information: the white rectangle represents protein coding genes (PCGs), the red rectangle represents ribosomal RNA genes (rRNAs), and the green rectangle represents transfer RNA genes (tRNAs). The introns are connected by line segments.

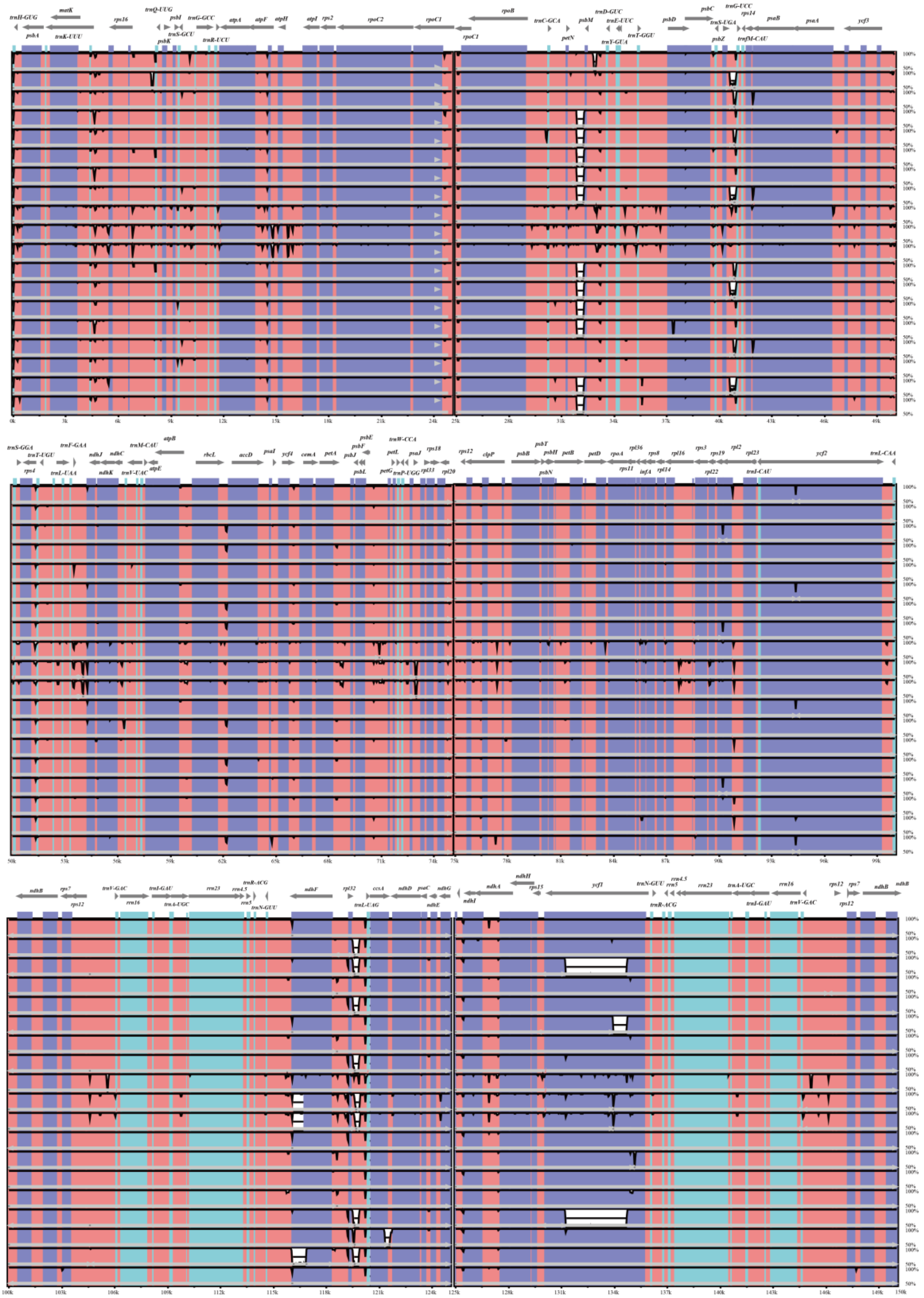

trnH-GUG
matK
psbA
trnK-UUU
rps16
trnQ-UUG
psbI
psbK
trnS-GCU
trnG-GCC
trnR-UCU
atpA
atpF
atpH
atpI
rps2
rpoC2
rpoC1
rpoB
trnC-GCA
petN
psbM
trnD-GUC
trnE-UUC
trnY-GUA
trnT-GGU
psbD
psbC
trnG-UCC
rps14
psbZ
trnS-UGA
trnfM-CAU
psaB
psaA
ycf3
trnS-GGA
trnT-UGU
rps4
trnF-GAA
trnL-UAA
ndhJ
ndhC
ndhK
trnM-CAU
trnV-UAC
atpE
atpB
rbcL
accD
psaI
ycf4
cemA
petA
psbJ
psbL
psbF
psbE
petL
petG
trnW-CCA
trnP-UGG
psaJ
rps18
rpl33
rpl20
rps12
clpP
psbB
psbT
psbH
psbN
petB
petD
rpoA
rpl36
rps11
rps8
infA
rpl14
rpl16
rps3
rps19
rpl22
rpl2
rpl23
trnI-CAU
ycf2
trnL-CAA
ndhB
rps7
trnV-GAC
rrn16
trnI-GAU
trnA-UGC
rrn23
trnR-ACG
rrn4.5
rrn5
trnN-GUU
ndhF
rpl32
trnL-UAG
ccsA
ndhD
psaC
ndhE
ndhG
ndhI
ndhA
ndhH
rps15
ycf1
0k
3k
6k
9k
12k
15k
18k
21k
24k
25k
28k
31k
34k
37k
40k
43k
46k
49k
50k
53k
56k
59k
62k
65k
68k
71k
74k
75k
78k
81k
84k
87k
90k
93k
96k
99k
100k
103k
106k
109k
112k
115k
118k
121k
124k
125k
128k
131k
134k
137k
140k
143k
146k
149k
150k
100%
50%

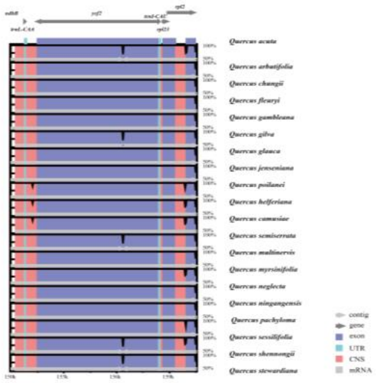

Figure S2: Sequence alignment of the chloroplast genomes of four *Quercus* section *Cyclobalanopsis* species. The *Q. acuta* was used as reference. The gray arrow above the map shows the location of the reference sequence gene, and the direction of the arrow is the forward or reverse direction of the gene. The position of the genome is shown on the horizontal axis at the bottom of each block. The alignment similarity percentages are shown on the right side of the map (vertical axis). Genome regions are color coded as exon, UTR, mRNA, and conserved non-coding sequences (CNS).

Table S1: Simple sequence repeats (SSRs) number in the chloroplast genomes of four *Quercus* section *Cyclobalanopsis* species. Abbreviations: LSC (Large Single Copy), SSC (Small Single Copy), IRs (Inverted Repeats), IGS (Intergenic Spacer), and GR (Gene Region).

Species	Repeat Type	Repeat Unit	No. of SSRs	Region			Location	
				LSC	SSC	IRs	IGS	GR
Q. poilanei	Mononucleotides	A/T	77	59	12	6	61	16
		C/G	5	5	0	0	3	2
	Dinucleotides	AG/CT	3	0	1	2	2	1
		AT/AT	12	12	0	0	8	4
	Trinucleotides	AAG/CTT	1	0	1	0	0	1
		AAT/ATT	6	4	2	0	3	3
	Tetranucleotides	AAAT/ATTT	6	4	2	0	3	3
		AATG/ATTC	1	1	0	0	1	0
		AATT/AATT	2	2	0	0	1	1
	Pentanucleotides	AAAAT/ATTTT	1	0	1	0	0	1
		AATAT/ATATT	0	0	0	0	0	0
		AATGC/ATTGC	2	0	0	2	2	0
	Mononucleotides	A/T	77	59	14	4	54	23
		C/G	5	4	1	0	2	3
	Dinucleotides	AG/CT	3	1	0	2	2	1
		AT/AT	14	13	1	0	11	3

Q. helferiana	Trinucleotides	AAG/CTT	1	0	1	0	0	1
		AAT/ATT	5	3	2	0	2	3
	Tetranucleotides	AAAT/ATTT	6	6	0	0	5	1
		AATG/ATTC	1	1	0	0	1	0
		AATT/AATT	2	2	0	0	1	1
	Pentanucleotides	AAAAT/ATTTT	1	0	1	0	0	1
		AATAT/ATATT	0	0	0	0	0	0
		AATGC/ATTGC	2	0	0	2	2	0
Q. camusiae	Mononucleotides	A/T	78	58	16	4	58	20
		C/G	4	3	1	0	1	3
	Dinucleotides	AG/CT	3	1	0	2	2	1
		AT/AT	14	13	1	0	11	3
	Trinucleotides	AAG/CTT	1	0	1	0	0	1
		AAT/ATT	7	4	1	2	3	4
	Tetranucleotides	AAAT/ATTT	7	6	1	0	5	2
		AATG/ATTC	1	1	0	0	1	0
		AATT/AATT	2	2	0	0	1	1
	Pentanucleotides	AAAAT/ATTTT	1	0	1	0	0	1
		AATAT/ATATT	2	1	1	0	1	1
		AATGC/ATTGC	2	0	0	2	2	0
	Hexanucleotide	ACCATG/ATGGTC	1	1	0	0	0	1
Q. semiserrata	Mononucleotides	A/T	78	58	16	4	58	20
		C/G	4	3	1	0	1	3
	Dinucleotides	AG/CT	3	1	0	2	2	1
		AT/AT	14	13	1	0	11	3
	Trinucleotides	AAG/CTT	1	0	1	0	0	1
		AAT/ATT	7	4	1	2	3	4
	Tetranucleotides	AAAT/ATTT	7	6	1	0	5	2
		AATG/ATTC	1	1	0	0	1	0
		AATT/AATT	2	2	0	0	1	1
	Pentanucleotides	AAAAT/ATTTT	1	0	1	0	0	1
		AATAT/ATATT	2	1	1	0	1	1
		AATGC/ATTGC	2	0	0	2	2	0
	Hexanucleotides	ACCATG/ATGGTC	1	1	0	0	0	1

Table S2a. Codon parameter characterization of chloroplast genome of *Q. poilanei*. Abbreviations: ENC (Effective Number of Codon).

Gene name	*Q. poilanei*									
	T3s	C3s	A3s	G3s	GC1 (%)	GC2 (%)	GC3 (%)	GC_all (%)	GC3s	ENc
psbA	0.5493	0.2368	0.2937	0.0811	49.44	43.5	31.64	41.53	0.272	40.77
matK	0.4848	0.1725	0.4419	0.1954	40.59	32.87	28.51	33.99	0.27	50.79
atpA	0.4523	0.1683	0.4276	0.1697	55.31	39.76	28.35	41.14	0.267	48.23
atpF	0.3836	0.1849	0.4444	0.2538	45.41	35.14	35.14	38.56	0.333	55.04
atpI	0.5142	0.1698	0.4115	0.0904	48.79	36.29	23.79	36.29	0.213	43.56

rps2	0.4921	0.1257	0.4477	0.1824	43.04	40.93	27.43	37.13	0.23	47.35
rpoC2	0.4446	0.1621	0.4612	0.1961	45.47	37.07	28.88	37.14	0.269	50.3
rpoC1	0.4596	0.1636	0.4366	0.2119	50.07	38.07	30.6	39.58	0.281	51.99
rpoB	0.4413	0.1385	0.4515	0.2264	50.37	37.62	29.8	39.26	0.275	50.09
psbD	0.4800	0.2233	0.3525	0.1478	52.26	43.22	34.75	43.41	0.305	48.25
rps14	0.3816	0.1316	0.4878	0.2208	46.53	47.52	30.69	41.58	0.281	38.42
psaB	0.4968	0.1581	0.3821	0.1785	48.84	43.13	30.88	40.95	0.264	49.68
psaA	0.4465	0.2	0.3922	0.1642	52.46	43.54	33.42	43.14	0.294	51.15
ycf3	0.4361	0.1805	0.437	0.2621	46.15	38.46	34.91	39.84	0.317	56.39
rps4	0.4843	0.1509	0.4663	0.1409	50.25	38.42	24.14	37.6	0.227	47.88
ndhJ	0.5078	0.1406	0.4128	0.2083	49.69	38.99	30.19	39.62	0.257	49.16
ndhK	0.4667	0.1385	0.4343	0.1761	44.59	45.89	27.71	39.39	0.248	53.28
ndhC	0.5400	0.08	0.4253	0.1644	48.76	34.71	24.79	36.09	0.18	45
atpE	0.4766	0.1495	0.3796	0.2021	52.24	40.3	30.60	41.04	0.276	51.34
atpB	0.4398	0.1794	0.4458	0.1337	55.91	41.88	27.66	41.82	0.255	45.42
rbcL	0.5064	0.1979	0.3746	0.1536	58.19	43.91	30.46	44.19	0.279	48.54
accD	0.5644	0.1710	0.374	0.1841	39.73	34.22	28.52	34.16	0.258	49.51
ycf4	0.4934	0.1711	0.3704	0.2018	43.24	40	31.89	38.38	0.282	50.57
cemA	0.478	0.2253	0.4151	0.1591	40.43	28.26	33.04	33.91	0.288	43.51
petA	0.4767	0.1977	0.358	0.2212	54.35	35.71	33.23	41.10	0.320	51.08
rps18	0.4896	0.1250	0.3579	0.2791	39.17	40.83	31.67	37.22	0.308	42.77
rpl20	0.4800	0.090	0.4348	0.2267	34.75	43.22	24.58	34.18	0.228	49.77
clpP1	0.4331	0.1592	0.4797	0.1493	58.88	38.07	28.93	41.96	0.245	54.63
psbB	0.4874	0.1724	0.3566	0.1773	54.81	46.37	32.02	44.40	0.283	49.81
petB	0.4722	0.1833	0.3742	0.1409	49.07	41.67	32.41	41.05	0.270	45.23
petD	0.4275	0.1304	0.5	0.1228	50.31	38.51	24.22	37.68	0.209	38.06
rpoA	0.4639	0.1559	0.51	0.1557	45.18	32.53	24.7	34.14	0.229	48.99
rps11	0.3802	0.1818	0.4087	0.1731	53.96	56.83	32.37	47.72	0.301	44.45
rps8	0.4286	0.1696	0.4434	0.1705	41.48	40.74	28.89	37.04	0.264	43.23
rpl14	0.4356	0.1683	0.5052	0.1098	53.66	37.40	23.58	38.21	0.218	44.68
rpl16	0.3551	0.1682	0.5179	0.1212	51.47	52.94	28.68	44.36	0.238	39.03
rps3	0.4329	0.189	0.5337	0.1156	50.45	34.23	24.77	36.49	0.224	48.34
rpl22	0.4405	0.1905	0.4651	0.1867	35.71	36.61	30.36	34.23	0.280	49.42
ndhF	0.5375	0.1072	0.4381	0.1422	35.37	35.11	22.61	31.03	0.186	43.04
ccsA	0.4596	0.1434	0.4741	0.1263	34.8	37.62	26.02	32.81	0.211	47.20
ndhE	0.5506	0.1461	0.3784	0.1148	40.2	34.31	24.51	33.01	0.206	51.93
ndhG	0.4717	0.1258	0.3731	0.2202	42.94	35.03	28.81	35.59	0.260	49.16
ndhI	0.5556	0.1556	0.4554	0.1031	43.37	36.14	23.49	34.34	0.197	44.27
ndhA	0.4317	0.1556	0.4639	0.128	45.05	37.64	25.27	35.99	0.230	47.05
ndhH	0.4498	0.1586	0.4772	0.1755	51.52	36.29	29.95	39.26	0.251	49.76
ycf1	0.473	0.1624	0.5387	0.1819	37.12	28.04	26.5	30.55	0.241	49.03
rps7	0.4167	0.15	0.5194	0.1186	53.21	45.51	24.36	41.03	0.215	45.45
ndhB	0.4338	0.21	0.4164	0.1384	41.29	38.75	32.09	37.38	0.282	49.06
ycf2	0.4394	0.2148	0.3989	0.2630	41.44	34.24	37.14	37.61	0.347	53.26

rpl2	0.4034	0.1888	0.4404	0.1735	50.91	48	30.91	43.27	0.291	51.66

Table S2b. Codon parameter characterization of chloroplast genome of *Q. helferiana*. Abbreviations: ENC (Effective Number of Codon).

Gene name	*Q. helferiana*									
	T3s	C3s	A3s	G3s	GC1 (%)	GC2 (%)	GC3 (%)	GC_all (%)	GC3s	ENc
psbA	0.5493	0.2368	0.2937	0.0811	49.44	43.5	31.64	41.53	0.272	40.77
matK	0.4836	0.1729	0.4377	0.202	40.59	32.67	28.91	34.06	0.274	50.89
atpA	0.4472	0.1709	0.4276	0.1723	55.31	39.76	28.74	41.27	0.271	48.9
atpF	0.3836	0.1849	0.4414	0.2519	45.41	35.68	35.14	38.74	0.333	55.58
atpI	0.5094	0.1745	0.4115	0.0904	48.79	36.29	24.19	36.42	0.218	43.86
rps2	0.4974	0.1257	0.4419	0.1812	43.88	40.93	27.43	37.41	0.23	48.78
rpoC2	0.4464	0.1603	0.4602	0.1976	45.33	37.07	28.81	37.07	0.268	49.99
rpoC1	0.4586	0.1639	0.4358	0.2137	50.22	37.92	30.75	39.63	0.282	51.88
rpoB	0.4413	0.1385	0.4515	0.2267	50.37	37.8	29.8	39.32	0.275	50.24
psbD	0.4867	0.22	0.3525	0.1435	52.26	43.22	34.18	43.22	0.299	48.67
rps14	0.3947	0.1184	0.4878	0.2208	46.53	46.53	29.7	40.92	0.271	39.32
psaB	0.4968	0.1597	0.386	0.172	48.71	43.13	30.61	40.82	0.261	49.7
psaA	0.4458	0.1997	0.3878	0.1701	52.33	43.68	33.69	43.23	0.298	51.27
ycf3	0.4361	0.1805	0.437	0.2621	46.15	38.46	34.91	39.84	0.317	56.39
rps4	0.4843	0.1509	0.4601	0.1477	50.25	38.42	24.63	37.77	0.232	48.02
ndhJ	0.5078	0.1406	0.4128	0.2083	49.06	38.99	30.19	39.41	0.257	50.15
ndhK	0.4667	0.1385	0.44	0.1698	44.59	45.89	27.27	39.25	0.243	52.99
ndhC	0.54	0.08	0.4253	0.1644	48.76	34.71	24.79	36.09	0.18	45
atpE	0.4766	0.1495	0.3796	0.2021	52.24	40.3	30.6	41.04	0.276	51.34
atpB	0.4373	0.1818	0.4458	0.1337	55.91	41.88	27.86	41.88	0.257	45.52
rbcL	0.509	0.1954	0.3775	0.1506	57.98	43.7	30.04	43.91	0.275	48.02
accD	0.561	0.1737	0.375	0.181	39.5	34.54	28.63	34.22	0.259	49.79
ycf4	0.4868	0.1776	0.3778	0.193	43.24	40	31.89	38.38	0.282	52.08
cemA	0.4835	0.2198	0.4151	0.1591	40.43	28.26	32.61	33.77	0.284	43.44
petA	0.4767	0.1977	0.3502	0.2291	54.35	36.02	33.85	41.41	0.326	51.89
rps18	0.4896	0.125	0.3579	0.2824	38.33	40.83	31.67	36.94	0.308	42.56
rpl20	0.48	0.09	0.4286	0.24	34.75	43.22	25.42	34.46	0.237	51.32
clpP1	0.4331	0.1592	0.4797	0.1493	58.88	38.07	28.93	41.96	0.245	54.63
psbB	0.4874	0.1701	0.3566	0.1802	54.81	46.37	32.02	44.4	0.283	49.66
petB	0.4722	0.1833	0.3742	0.1409	49.07	41.67	32.41	41.05	0.27	45.23
petD	0.4348	0.1232	0.4839	0.1404	50.31	38.51	24.84	37.89	0.216	37.95
rpoA	0.4601	0.1597	0.51	0.1557	45.18	32.53	25	34.24	0.232	48.95
rps11	0.3802	0.1818	0.4052	0.1714	53.96	57.55	32.37	47.96	0.301	43.91
rps8	0.4375	0.1607	0.4434	0.1705	41.48	40.74	28.15	36.79	0.256	43.23
rpl14	0.4356	0.1683	0.5052	0.1098	53.66	37.4	23.58	38.21	0.218	44.68
rpl16	0.3611	0.1667	0.5133	0.1212	51.47	52.94	27.94	44.12	0.236	39.09
rps3	0.4329	0.189	0.5337	0.1156	50.45	34.23	24.77	36.49	0.224	48.34

ndhF	0.5422	0.1099	0.4361	0.1384	35.03	35.03	22.48	30.85	0.186	42.66
ccsA	0.4613	0.1439	0.4655	0.1368	34.8	37.62	26.65	33.02	0.218	48.42
ndhE	0.5618	0.1348	0.3784	0.1148	40.2	34.31	23.53	32.68	0.196	51.93
ndhG	0.478	0.1195	0.3731	0.2202	42.94	35.03	28.25	35.4	0.254	49.61
ndhI	0.5556	0.1556	0.4554	0.1031	44.58	36.14	23.49	34.74	0.197	43.09
ndhA	0.4286	0.1587	0.457	0.136	45.05	37.64	26.1	36.26	0.239	47.55
ndhH	0.4513	0.1591	0.4772	0.1755	51.02	36.04	29.95	39	0.251	50.03
ycf1	0.4713	0.1628	0.5416	0.1802	36.8	27.93	26.4	30.38	0.24	48.62
rps7	0.4167	0.15	0.5194	0.1186	53.21	45.51	24.36	41.03	0.215	45.45
ndhB	0.4338	0.21	0.4164	0.1384	41.29	38.75	32.09	37.38	0.282	49.06
ycf2	0.4402	0.2143	0.3997	0.2617	41.48	34.24	37.05	37.59	0.346	53.31
rpl2	0.4034	0.1888	0.4404	0.1735	50.91	48	30.91	43.27	0.291	51.66

Table S2c. Codon parameter characterization of chloroplast genome of *Q. camusiae* and *Q. semiserrata*. Abbreviations: ENC (Effective Number of Codon).

Gene name	***Q. camusiae* and *Q. semiserrata***									
	**T3s**	**C3s**	**A3s**	**G3s**	**GC1 (%)**	**GC2 (%)**	**GC3 (%)**	**GC_all (%)**	**GC3s**	**ENc**
psbA	0.5493	0.2368	0.2937	0.0811	49.44	43.5	31.64	41.53	0.272	40.77
matK	0.4791	0.1791	0.438	0.1967	40.83	32.94	29.19	34.32	0.277	51.36
atpA	0.4497	0.1683	0.4276	0.1719	55.51	39.76	28.54	41.27	0.269	48.49
atpF	0.3836	0.1849	0.4514	0.2462	45.41	34.59	34.59	38.2	0.328	56.03
atpI	0.5189	0.1698	0.4063	0.0898	49.19	36.29	23.79	36.42	0.213	43.7
rps2	0.4921	0.1257	0.4503	0.1837	43.04	40.51	27.43	36.99	0.23	47.57
rpoC2	0.4452	0.1608	0.4594	0.1978	45.4	37.28	28.74	37.14	0.269	50.22
rpoC1	0.4632	0.1618	0.4327	0.2132	50.07	38.21	30.45	39.58	0.281	51.59
rpoB	0.442	0.1383	0.4522	0.2248	50.37	37.52	29.61	39.17	0.274	49.78
psbD	0.4867	0.22	0.3484	0.1478	52.26	43.22	34.46	43.31	0.302	48.69
rps14	0.3947	0.1184	0.4878	0.2208	46.53	47.52	29.7	41.25	0.271	38.42
psaB	0.5016	0.155	0.384	0.1742	48.71	43.13	30.34	40.73	0.258	49.48
psaA	0.4465	0.2	0.3866	0.1705	52.33	43.54	33.82	43.23	0.298	51.85
ycf3	0.4361	0.1805	0.4407	0.2647	45.56	38.46	34.91	39.64	0.317	55.98
rps4	0.478	0.1572	0.4724	0.1342	50.25	38.42	24.14	37.6	0.227	49.16
ndhJ	0.5039	0.1395	0.4091	0.2165	50.31	38.99	30.19	39.83	0.262	49.9
ndhK	0.4667	0.1385	0.44	0.1698	44.59	45.89	27.27	39.25	0.243	52.99
ndhC	0.54	0.08	0.4253	0.1667	47.93	34.71	24.79	35.81	0.18	45.21
atpE	0.4766	0.1495	0.3796	0.2021	52.24	40.3	30.6	41.04	0.276	51.34
atpB	0.4447	0.1744	0.4458	0.1337	55.91	41.88	27.25	41.68	0.251	45.1
rbcL	0.5064	0.1979	0.3775	0.1506	57.98	43.7	30.25	43.98	0.277	48.15
accD	0.56	0.1765	0.3764	0.1795	39.39	34.8	28.87	34.35	0.26	49.73
ycf4	0.4868	0.1776	0.3778	0.1913	43.78	40	31.89	38.56	0.282	51.94
cemA	0.4754	0.2295	0.4177	0.1527	40.43	28.26	33.04	33.91	0.288	43.56
petA	0.4767	0.1977	0.3541	0.2267	54.04	35.71	33.54	41.1	0.323	51.32
rps18	0.4896	0.125	0.3474	0.2941	38.33	40.83	32.5	37.22	0.316	43.05

rpl20	0.48	0.08	0.4348	0.24	34.75	42.37	24.58	33.9	0.228	50.52
clpP1	0.4331	0.1592	0.4797	0.1493	58.88	38.07	28.93	41.96	0.245	54.63
psbB	0.4874	0.1701	0.3539	0.1831	54.81	46.37	32.22	44.47	0.285	49.72
petB	0.4722	0.1778	0.3804	0.1409	49.07	41.67	31.94	40.9	0.265	45.3
petD	0.442	0.1159	0.5	0.1228	50.31	38.51	22.98	37.27	0.196	37.1
rpoA	0.4601	0.1597	0.51	0.1557	45.18	32.53	25	34.24	0.232	48.95
rps11	0.3802	0.1818	0.4052	0.1714	53.96	57.55	32.37	47.96	0.301	43.91
rps8	0.4425	0.1593	0.4381	0.1724	41.48	40.74	28.15	36.79	0.256	42.19
rpl14	0.4356	0.1683	0.5052	0.1098	53.66	37.4	23.58	38.21	0.218	44.68
rpl16	0.3551	0.1682	0.5179	0.1212	51.47	52.94	28.68	44.36	0.238	39.03
rps3	0.4294	0.1902	0.5367	0.1156	50.45	34.23	25.23	36.64	0.225	48.41
ndhF	0.5354	0.1108	0.4417	0.1361	35.51	34.98	22.56	31.02	0.186	43.08
ccsA	0.4615	0.1392	0.4721	0.1316	35	37.5	25.94	32.81	0.211	47.5
ndhE	0.5618	0.1348	0.3919	0.0984	40.2	34.31	22.55	32.35	0.186	51.93
ndhG	0.481	0.1203	0.3731	0.2202	42.94	34.46	28.25	35.22	0.254	47.86
ndhI	0.5556	0.1481	0.4643	0.1031	43.98	36.14	22.89	34.34	0.191	42.1
ndhA	0.4317	0.1556	0.4639	0.128	45.05	37.64	25.27	35.99	0.23	47.05
ndhH	0.4628	0.1553	0.4789	0.1639	50.76	36.04	28.93	38.58	0.24	49.35
ycf1	0.4743	0.1629	0.532	0.1877	37.16	27.95	27	30.7	0.245	48.99
rps7	0.4167	0.15	0.5194	0.1186	53.21	45.51	24.36	41.03	0.215	45.45
ndhB	0.4315	0.21	0.4164	0.1415	41.29	38.75	32.29	37.44	0.284	49.32
ycf2	0.44	0.2142	0.3997	0.2624	41.48	34.28	37.05	37.61	0.346	53.29
rpl2	0.4034	0.1888	0.4404	0.1735	50.91	48	30.91	43.27	0.291	51.66

Table S3: The relative synonymous codon usage in four chloroplast genomes of *Quercus* section *Cyclobalanopsis*.

Types	*Q. poilanei*	*Q. helferiana*	*Q. camusiae*	*Q. semiserrata*
GCA	1.1069	1.0995	1.1164	1.1164
GCC	0.611	0.6184	0.6073	0.6073
GCG	0.5031	0.4955	0.4836	0.4836
GCU	1.779	1.7866	1.7927	1.7927
UGC	0.5565	0.5526	0.5487	0.5487
UGU	1.4435	1.4474	1.4513	1.4513
GAC	0.3803	0.3807	0.3818	0.3818
GAU	1.6197	1.6193	1.6182	1.6182
GAA	1.5284	1.5221	1.5154	1.5154
GAG	0.4716	0.4779	0.4846	0.4846
UUC	0.6732	0.6691	0.6727	0.6727
UUU	1.3268	1.3309	1.3273	1.3273
GGA	1.5065	1.4989	1.5058	1.5058
GGC	0.4849	0.4939	0.474	0.474
GGG	0.703	0.7018	0.6936	0.6936
GGU	1.3056	1.3054	1.3266	1.3266
CAC	0.4587	0.4568	0.4523	0.4523

CAU	1.5413	1.5432	1.5477	1.5477
AUA	0.9503	0.9496	0.9555	0.9555
AUC	0.5765	0.5748	0.5733	0.5733
AUU	1.4732	1.4756	1.4713	1.4713
AAA	1.5121	1.5159	1.508	1.508
AAG	0.4879	0.4841	0.492	0.492
CUA	0.8034	0.8	0.793	0.793
CUC	0.3932	0.3943	0.3894	0.3894
CUG	0.4017	0.3915	0.4036	0.4036
CUU	1.2107	1.217	1.2136	1.2136
UUA	1.9604	1.9546	1.9668	1.9668
UUG	1.2306	1.2426	1.2335	1.2335
AAC	0.4513	0.4535	0.4575	0.4575
AAU	1.5487	1.5465	1.5425	1.5425
CCA	1.0835	1.0734	1.1005	1.1005
CCC	0.8162	0.8183	0.823	0.823
CCG	0.5871	0.6065	0.5789	0.5789
CCU	1.5131	1.5018	1.4976	1.4976
CAA	1.5448	1.5341	1.5422	1.5422
CAG	0.4552	0.4659	0.4578	0.4578
AGA	1.8377	1.8465	1.8215	1.8215
AGG	0.6457	0.6455	0.6505	0.6505
CGA	1.346	1.3561	1.3511	1.3511
CGC	0.4023	0.3953	0.4103	0.4103
CGG	0.447	0.4504	0.4554	0.4554
CGU	1.3212	1.3061	1.3111	1.3111
AGC	0.3758	0.3682	0.3699	0.3699
AGU	1.2404	1.234	1.2358	1.2358
UCA	1.1758	1.1895	1.1951	1.1951
UCC	1.0101	1.0074	0.9959	0.9959
UCG	0.5859	0.5624	0.5569	0.5569
UCU	1.6121	1.6386	1.6463	1.6463
ACA	1.2348	1.2295	1.2167	1.2167
ACC	0.7113	0.7066	0.7038	0.7038
ACG	0.4396	0.4431	0.4533	0.4533
ACU	1.6144	1.6208	1.6262	1.6262
GUA	1.5501	1.5556	1.5354	1.5354
GUC	0.4625	0.4627	0.4682	0.4682
GUG	0.5528	0.5574	0.5662	0.5662
GUU	1.4345	1.4244	1.4301	1.4301
UAC	0.4264	0.4215	0.4235	0.4235
UAU	1.5736	1.5785	1.5765	1.5765

Table S4: Likelihood ratio test (LRT) and positive selection sites under different site models of PCGs of four *Quercus* section *Cyclobalanopsis*.

Genes	Model Comparison	df	ΔlnL	2ΔlnL	LRT (P-value)	Positively selected sites (BEB: *: P>95%; **: P>99%)
atpF	M0 vs M3	4	36.484478	72.968956	5.36E-15	
	M1 vs M2	2	21.965641	43.931282	2.89E-10	17A (0.621),49S (0.996**),50D (0.993**),52N (0.994**),54R (1.000**),104N (0.545)
	M7 vs M8	2	22.022258	44.044516	2.73E-10	17A (0.674),49S (0.998**),50D (0.997**),52N (0.998**),54R (1.000**),104N (0.598)
ndhA	M0 vs M3	4	5.597318	11.194636	0.024461501	
	M1 vs M2	2	3.645198	7.290396	0.026116238	34L (0.526),280I (0.640), 281S (0.851)
	M7 vs M8	2	3.701774	7.403548	0.024679706	34L (0.592),280I (0.693),281S (0.897)
ndhD	M0 vs M3	4	13.663614	27.327228	1.71E-05	
	M1 vs M2	2	9.273251	18.546502	9.39E-05	75D (0.939),201V (0.572),364T (0.592),408Q (0.609),409N (0.937),499L (0.990*)
	M7 vs M8	2	6.203671	12.407342	0.002021994	75D (0.966*),201V (0.657),364T (0.672),408Q (0.688),409N (0.966*),499L (0.996**)
ndhF	M0 vs M3	4	5.786362	11.572724	0.020828209	
	M1 vs M2	2	4.261784	8.523568	0.014097131	487N (0.686),745K (0.945)
	M7 vs M8	2	4.16729	8.33458	0.015494193	59S(0.569),222N(0.564),376D(0.560),383L(0.548),424T(0.560),457Y(0.569),487N(0.788),489P(0.576),571Q(0.608),575G(0.572),582S(0.606),652V(0.543),741L(0.565),743F(0.598),745K (0.972*)
ndhK	M0 vs M3	4	5.049127	10.098254	0.038804734	
	M1 vs M2	2	3.672552	7.345104	0.025411537	15Q (0.777)
	M7 vs M8	2	3.691397	7.382794	0.024937141	15Q (0.882)
petB	M0 vs M3	4	4.761273	9.522546	0.049286033	
	M1 vs M2	2	3.725946	7.451892	0.0240903	1V (0.791)
	M7 vs M8	2	3.730301	7.460602	0.023985615	1V (0.891)
petD	M0 vs M3	4	21.745095	43.49019	8.19E-09	
	M1 vs M2	2	11.904026	23.808052	6.76E-06	1M (0.938),2G (0.999**),3V (0.989*)
	M7 vs M8	2	11.999172	23.998344	6.15E-06	1M (0.964*),2G (1.000**),3V (0.994**)
rbcL	M0 vs M3	4	10.081759	20.163518	0.000463614	
	M1 vs M2	2	7.528338	15.056676	0.000537631	219V (0.960*),328A (0.514)
	M7 vs M8	2	7.764799	15.529598	0.000424415	219V (0.982*),262A (0.593),328A (0.623)
rpl22	M0 vs M3	4	7.293626	14.587252	0.005638491	
	M1 vs M2	2	0.55532	1.11064	0.573888587	6K (0.965*)
	M7 vs M8	2	6.775851	13.551702	0.001140999	6K (0.995**),7R (0.561),9I(0.502),10E(0.541),15C (0.577),37S (0.721),50E (0.526),53L (0.651),59E (0.533)
ycf1	M0 vs M3	4	105.976296	211.952592	1.01E-44	
	M1 vs M2	2	83.329584	166.659168	6.46E-37	3L(0.541),163K(0.532),242V(0.526),249E(0.949),400R(0.565),449S(0.558),499C(0.530),506L(0.578),533F(0.509),542R(0.585),573F(0.556),592K(0.528),598L(0.543),650L(0.503),7

						32P(0.599),743F(0.946),777K(0.553),978F(0.523),1249R(0.561),1271Y(0.511),1366Y(0.519),1405S(0.532),1489Q(1.000**),1490G(1.000**),1491F(1.000**),1492F(0.996**),1493F(0.995**),1518F(0.511),1543V(0.508),1561P(0.512),1570L(0.556),1575Q(0.519),1589P(0.547),1597V(0.524),1643Q(0.519),1684D(0.516),1803T(0.554),1814C(0.591)
	M7 vs M8	2	83.756869	167.513738	4.22E-37	3L(0.698),163K(0.690),242V(0.685),249E(0.981*),346D(0.621),347K(0.635),400R(0.720),449S(0.713),499C(0.688),506L(0.731),533F(0.669),542R(0.737),555K(0.626),556M(0.645),573F(0.712),592K(0.685),598L(0.700),618D(0.643),650L(0.662),732P(0.749),743F(0.980*),767I(0.635),777K(0.709),917R(0.651),957I(0.638),978F(0.681),1061I(0.640),1084R(0.657),1189S(0.651),1249R(0.716),1271Y(0.670),1366Y(0.678),1405S(0.690),1489Q(1.000**),1490G(1.000**),1491F(1.000**),1492F(0.999**),1493F(0.999**),1518F(0.670),1540D(0.632),1543V(0.668),1561P(0.672),1570L(0.712),1574N(0.623),1575Q(0.678),1589P(0.704),1597V(0.683),1643Q(0.678),1684D(0.675),1803T(0.710),1814C(0.742)
ycf3	M0 vs M3	4	40.067163	80.134326	1.63E-16	
	M1 vs M2	2	30.357823	60.715646	6.54E-14	103L(0.890),118R(1.000**),119G(0.988*),120E(1.000**),121Q(0.991**)
	M7 vs M8	2	30.36522	60.73044	6.49E-14	103L(0.939),118R(1.000**),119G(0.995**),120E(1.000**),121Q(0.996**)
